# Heparin-Mediated Extracorporeal Low-Density Lipoprotein Precipitation Apheresis for Treating Peripheral Arterial Disease in Patients with Chronic Kidney Disease

**DOI:** 10.3390/jcm13041121

**Published:** 2024-02-16

**Authors:** Stefania Rotella, Loreto Gesualdo, Marco Fiorentino

**Affiliations:** Nephrology, Dialysis and Transplantation Unit, Department of Precision and Regenerative Medicine and Ionian Area (DiMePRE-J), University of Bari Aldo Moro, 70121 Bari, Italy; s.rotella@studenti.uniba.it (S.R.); loreto.gesualdo@uniba.it (L.G.)

**Keywords:** peripheral arterial disease, heparin-mediated extracorporeal low-density lipoprotein precipitation, chronic kidney disease

## Abstract

Patients with chronic kidney disease (CKD), particularly those with end-stage renal disease (ESRD), have a high prevalence of cardiovascular disease and peripheral arterial disease (PAD). Medical treatment is mainly based on risk factor management, and the surgical approach remains the gold standard treatment in specific conditions. Heparin-mediated extracorporeal low-density lipoprotein precipitation (H.E.L.P.) apheresis is effective in reducing circulating lipoprotein, fibrinogen, inflammatory mediators and procoagulant factors, thereby reducing cardiovascular risk in patients with familial hypercholesterolemia and hypertriglyceridemia. These activities may be effective in reducing symptoms and ischemic vascular lesions even in patients with severe PAD. We reported the application of a treatment protocol with H.E.L.P. apheresis in an ESRD patient with severe PAD without clinical improvement after severe revascularization who was not suitable for further surgical approaches, despite normal LDL cholesterol and lipoprotein (a). The H.E.L.P. protocol was characterized by an intensive first phase with weekly treatments followed by a single session every 10–15 days for 6 months of treatment. The overall clinical condition, foot lesions and walking distance improved significantly after the first 2 months of treatment, and foot amputation was avoided. Here, we review the main pathogenetic mechanisms through which LDL apheresis improves microcirculation and clinical outcomes. Its wider application may represent an optimal therapeutic option for patients unresponsive to standard treatment.

## 1. Introduction

Patients with chronic kidney disease (CKD) and those with end-stage renal disease (ESRD) requiring chronic hemodialysis are characterized by a high risk of cardiovascular disease (CVD) and worse prognosis [1,2]. Several risk factors for CVD in this specific population (diabetes, hypertension, hyperlipidemia, smoking, uremia, inflammation) significantly increase the risk of developing peripheral arterial disease (PAD), a serious clinical condition characterized by the progressive occlusion of peripheral arterial vessels [3]. PAD lesions are more distributed in lower limb arteries, with a severely stenotic lumen and vascular calcification in the absence of collateral circulation. The diagnosis of PAD is usually performed using the ankle brachial pressure index (ABI) associated with a lower limb Doppler ultrasound evaluation. The use of angiography and computerized tomography is typically reserved for more complicated patients [4]. Different classifications of vascular lesions have been proposed in order to stage the severity of the disease based on clinical presentation and symptoms. PAD classification according to the Leriche–Fontaine system includes the following four stages: stage I for asymptomatic and occasional reports of incomplete vessel obstruction, stage II characterized by mild intermittent claudication (stage IIa with a walking distance >200 m, stage IIb with a walking distance <200 m), stage III characterized by rest pain, and stage IV with the occurrence of different degrees of necrotic lesions [4]. Limited data on the prevalence of PAD among patients with ESRD have been reported in the literature to date; data from the PAD Awareness, Risk and Treatment: New Resources for Survival (PARTNERS) Study reported an overall prevalence of PAD in approximately 30% of the population [5]. Similarly, the Dialysis Outcomes and Practice Patterns Study (DOPPS) described PAD prevalence ranging from 25% to 46% between dialysis centers [6]. The “gold standard” for PAD treatment remains the surgical approach (angioplasty procedures, stent placement, peripheral artery bypass surgery) associated with medical therapy, including smoking cessation, blood pressure control, lipid-lowering therapies, antiplatelet drugs and cilostazol [3]; however, both traditional and minimally invasive procedures are often ineffective with a high risk of perioperative morbidity [3,7]. Low-density lipoprotein (LDL) apheresis is an extracorporeal treatment used in patients with severe dyslipidemia, such as familial hypercholesterolemia, at high risk of atherosclerotic disease, with the goal of reducing cardiovascular events [8,9,10]. Among the several techniques available for lipoprotein apheresis, heparin-mediated extracorporeal LDL precipitation (H.E.L.P.) apheresis represents an extracorporeal procedure able to remove plasma LDL, very low-density lipoprotein (VLDL), fibrinogen and lipoprotein (a) [Lp(a)], as well as inflammatory mediators [11]; these mechanisms of action represent the rationale for using such techniques as adjuvant therapy for PAD, reducing blood viscosity and atherosclerosis progression risk [12]. Here, we aim to review the application of lipoprotein apheresis for treating PAD, reporting the case of an ESRD patient with severe PAD treated with H.E.L.P. apheresis, with significant clinical improvement and foot amputation avoided. We then discuss the main evidence on the ability of LDL apheresis to target specific pathways (inflammation, coagulation, endothelial cells, vascular tone and oxidative stress) and improve clinical outcomes in PAD.

## 2. Case Report

**Figure 1:**
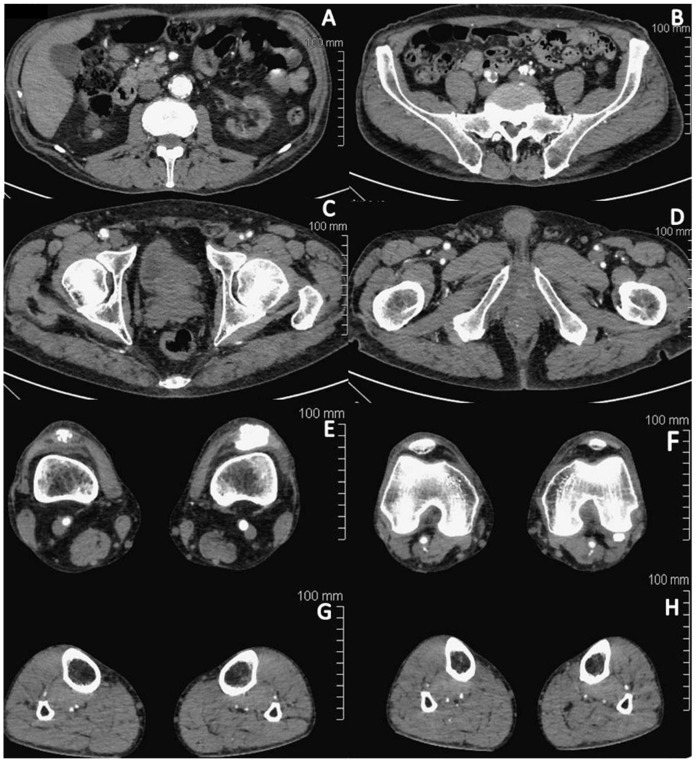
Angio-CT scan of the vascular axis in the case patient. (**A**) abdominal aorta with severe calcification and thrombotic plaques; (**B**) common iliac arteries with diffuse concentric parietal thrombotic apposition; (**C**) femoral arteries with diffuse parietal fibrocalcific apposition with irregular lumen; (**D**) femoral arteries with reduced lumen, particularly on the left side. (**E**) On the right side, pervious and diffusely atheromatic femoral popliteal axis. On the left, diffuse thrombotic apposition of the superficial femoral artery with presence of stenting to the popliteal axis. (**F**–**H**) Posterior tibial and interosseous arteries appear threadlike and tenuously opacified, especially in distal sections.

A 75-year-old male patient with ESRD treated with hemodialysis since September 2022 was referred to our center in February 2023 with clinically significant lower limb ulcers, severe pain and functional impairment associated with a marked inability to ambulate. In addition, his medical history reported a previous smoking habit, chronic hypertension in pharmacological treatment (amlodipine), chronic obstructive pulmonary disease (COPD) and a recent surgical procedure for bowel perforation. In 2017, chronic bilateral obliterative arteriopathy was diagnosed, and left superficial femoral artery revascularization was performed. In the following years, several revascularization procedures were performed without success or clinical improvement. In December 2022, the patient’s clinical conditions worsened, characterized by aggravated pain associated with edema and hyperemic skin of the lower limbs and gradual functional impairment. An angio-CT scan of the vascular axis was performed, which revealed diffuse calcification on the abdominal aorta with several atherosclerotic plaques and a filiform right posterior tibial artery; the left superficial femoral artery was characterized by diffuse thrombotic formation, with a vascular stent located in the third distal part of the artery (Figure 1). Severe atherosclerosis characterized the left anterior tibial artery, not visible at its distal tract. His treatment was based on antihypertensive drugs, bilastine at a dose of 20 mg/day and warfarin (previous atrial fibrillation episode), while statins were suspended due to patient intolerance.

After 3 months (February 2023), ulcers of the extremities appeared and were classified as grade III and IV, according to the Rutherford and Fontaine classifications, respectively. Despite the worsening of the patient’s clinical condition and instrumental information, revascularization was not likely to be feasible, and there were no indications for a vascular surgical procedure. The LDL cholesterol, HDL cholesterol, Lp(a) and fibrinogen values were 107 mg/dL, 40 mg/dL, 27.3 mg/dL and 432 mg/dL, respectively; the hematocrit value was 34.1%. D-dimers, prothrombin time (PT) and activated partial thromboplastin time (aPTT) were within the normal range. Because of the further worsening of the patient’s clinical condition and inability (significant reduction in the walking distance), despite the fact that no severe elevations in LDL cholesterol and Lp(a) levels were detected, we decided to treat the patient with H.E.L.P. apheresis. The apheresis procedure was performed as briefly summarized. Blood was drawn using an arteriovenous fistula of hemodialysis for vascular access. Four different phases characterize H.E.L.P. treatment: first, the cellular components were separated from the plasma using a plasma filter. Then, the plasma was acidified with an acetate–heparin buffer to induce fibrinogen and cholesterol precipitation in an acid solution (pH 4.85). Finally, the excess heparin was removed using a specific anion-exchange filter, and the pH was restored using a hemodialysis filter. The treated plasma was mixed with the cellular fraction and returned to the patient, whereas the precipitated lipoproteins and fibrinogen were discarded.

**Figure 2:**
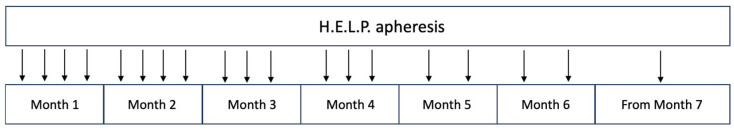
Treatment protocol. Legend: H.E.L.P, heparin-mediated extracorporeal LDL precipitation.

As reported in Figure 2, we started with a weekly interval between therapies, with 8 sessions in the first 2 months. We then switched to a single session every 10 days in the following 2 months (total 6 treatments), and then, a treatment on alternative weeks in the following 2 months (overall study treatment with 18 sessions in the 6-month period).

**Figure 3:**
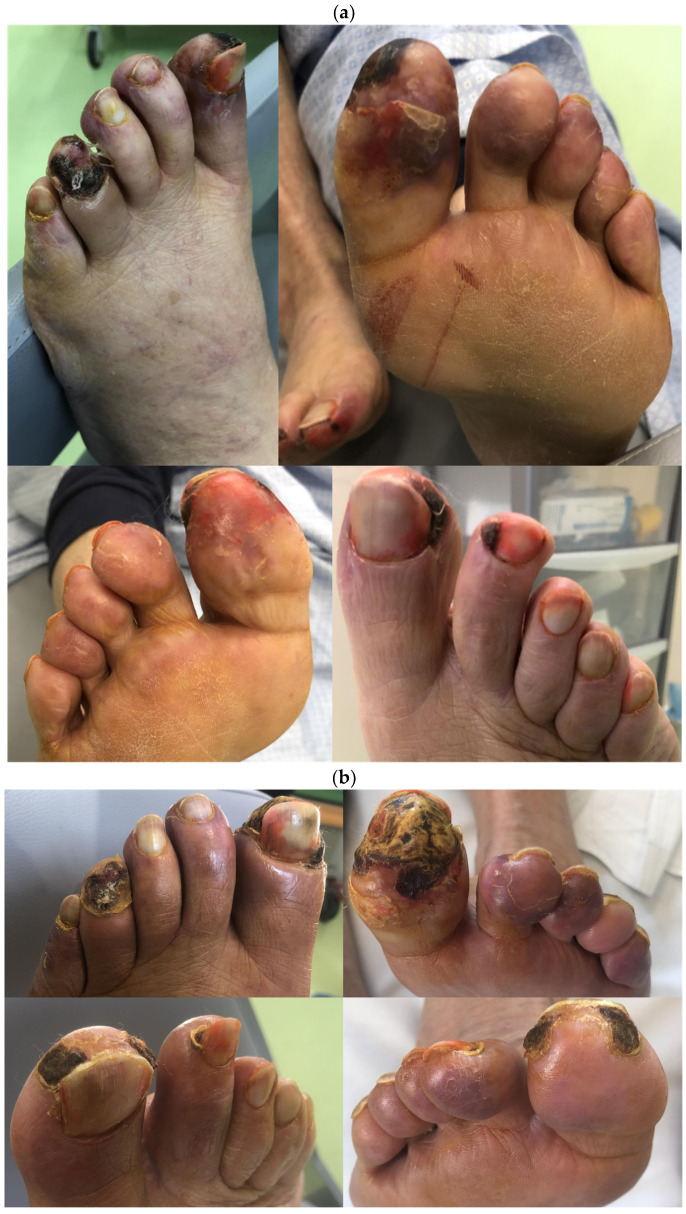

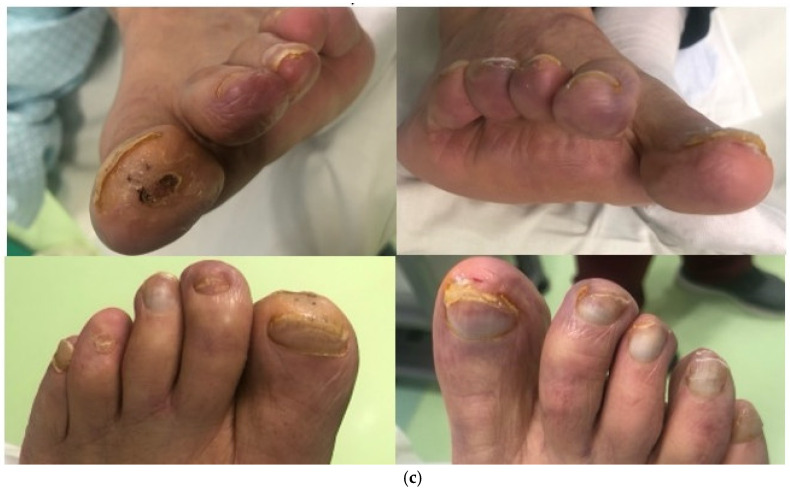
Ischemic foot lesions before treatment initiation (**a**), after 2 months (**b**) and after 6 months of treatment with H.E.L.P. apheresis (**c**).

Despite the advanced lesions presented at the start of treatment (Figure 3a), the patient’s condition improved soon after the first sessions. The initial symptoms, such as itching and uremic pain of the lower extremities, disappeared after the first two months of treatment (eight sessions), and the patient acquired total functional autonomy with a significant increase in walking distance. We observed a significant improvement in microvascular circulation with tissue regeneration and impressive improvements in foot trophism, as shown in Figure 3b. Table 1 summarizes the main parameters monitored during the treatment period. Ultrasound examination and vascular surgery consultation were performed 3 months later, confirming substantial improvements in lower limb microcirculation along with a slight improvement in ABI (on the left side, from 0.75 to 0.8; on the right side from 0.85 to 0.88). The patient proceeded with a single H.E.L.P. session every 15 days, with constant and significant improvement in the lesions. In September 2023, complete resolution of the lesions presented on the right foot was reported, whereas lesions on the left foot continued to improve with granulation tissue formation, and the necrotic part was almost lost. The patient continued a single monthly treatment after the 6-month protocol. In October 2023, examination of the lesion on the left foot clearly showed further improvement, characterized by the loss of the necrotic part (Figure 3c). The patient was clinically and functionally stable, with no symptoms related to limb ischemia. No significant side effects associated with H.E.L.P. procedures were reported during the study period.

## 3. Discussion

According to the European Society of Cardiology (ESC) guidelines, chronic limb-threatening ischemia management requires adequate risk factor control, constant wound care with antibiotic treatment in the case of superimposed infections, pain control, statins, vasodilatory agents and antithrombotic drugs. In severe cases that are unresponsive to medical treatment, a surgical approach based on angioplasty, peripheral artery stent placement or peripheral artery bypass surgery can be performed, leading to adequate tissue perfusion and clinical improvements. However, when revascularization is not feasible, or it is not possible to recover adequate vascularization, PAD progresses, potentially leading to amputation [3,13]. The American Society of Apheresis (ASFA) guidelines suggest lipoprotein apheresis as a possible treatment in patients with PAD, especially in those patients not suitable for a surgical approach or with severe pain-related symptoms and vascular lesion progression despite revascularization (Category II, Grade 1B) [14]. The rationale for this approach is strongly related to the pathophysiological processes of PAD, based on endothelial dysfunction, thrombogenesis and atherosclerosis. In addition, inflammatory mediators may stimulate atherosclerosis by upregulating the expression of adhesion molecules and chemokines, with consequent endothelial dysfunction [15]. Finally, high levels of fibrinogen induce blood hyperviscosity with platelet aggregation that increases the risk of atherosclerosis [16]. Lipoprotein apheresis is a unique approach able to remove lipoproteins, LDL cholesterol, Lp(a), fibrinogen, inflammatory mediators and coagulative factors, thereby reducing blood hyperviscosity and improving microcirculation and tissue perfusion [15]. In addition, lipoprotein apheresis significantly reduces the levels of circulating chemokines, tissue factor and CD40 ligands, which are key players in inflammation-related atherosclerosis [9,15].

### 3.1. Atherosclerosis and Lipoprotein Apheresis

**Figure 4:**
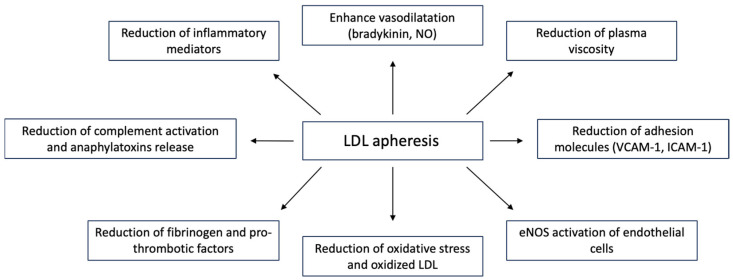
Main therapeutic effects of LDL apheresis. Legend: ICAM-1, intercellular adhesion molecule-1; LDL, low-density lipoprotein; NO, nitric oxide; VCAM-1, vascular adhesion molecule-1.

Although widely applied in the setting of severe familial hypercholesterolemia, extracorporeal LDL apheresis has been used in treating severe conditions linked to atherosclerosis and PAD [17]. LDL apheresis not only improves clinical symptoms, but is also associated with sustained improved long-term outcomes. Several mechanisms have been proposed to explain the potential role of LDL apheresis in atherosclerosis and PAD, and they are summarized in Figure 4. One of the key players in the atherosclerotic process is inflammation, and LDL apheresis can specifically target the inflammatory response at different levels [18]. CKD patients are characterized by chronic inflammation, particularly those on chronic hemodialysis. Several proinflammatory and anti-inflammatory cytokines can be modulated during LDL apheresis, thus justifying the ability of such treatment to affect inflammation-related atherosclerosis [19]. Overall, an increase in the anti-inflammatory cytokine interleukin-1 receptor antagonist (IL-1ra) and a decrease in the proinflammatory ones, such as interferon-γ, tumor necrosis factor-a (TNF-α) and regulated on activation, normal T cell expressed and secreted (RANTES), was reported by Hovland et al., when comparing the inflammatory response induced by three different LDL apheresis columns: DL-75 (whole-blood adsorption), LA-15 (plasma adsorption) and EC-50W (plasma filtration) [20]. In detail, RANTES is a cytokine produced by T cells and macrophages that plays a pivotal role in the inflammatory response as it can interact with P-selectin, inducing the attraction and infiltration of monocytes/macrophages into atherosclerotic lesions. Stefanutti et al. described the ability of LDL apheresis to decrease proinflammatory TNF-α and IL-1a in six patients with homozygous familial hypercholesterolemia. The authors also reported a significant reduction in IL-4 and IL-10 levels after apheresis, whereas a nonsignificant increase in IL-1ra was observed [21]. The same authors studied LDL apheresis in another group of patients with severe dyslipidemia and pre-existing angiographically who demonstrated coronary heart disease (CHD); lipoprotein apheresis was able to induce anti-inflammatory and anti-atherogenic action, with profound modulation of the cytokine profile (increase in RANTES levels, decrease in plasma macrophage inflammatory protein 1α (MIP-1α), macrophage inflammatory protein 1β (MIP-1β), monocyte chemoattractant protein-1 (MCP-1), TNF-α, IFN-γ, IL-1a, IL-1b and IL-6) [22].

In addition to cytokine modulation, a further burden to the link between inflammation and atherosclerosis is given by the activation and amplification of the alternative pathway; in fact, the release of the anaphylatoxins C3a and C5a is important during atherosclerosis, because they could be associated with atherosclerotic plaque instability and progression [23]. Although all kinds of extracorporeal treatments may stimulate complement activation due to direct interaction with biomaterials, several studies have shown the ability of different LDL apheresis techniques to adsorb the anaphylatoxins C3a and C5a [24,25,26]. In line with these results, a reduction in C-reactive protein (CRP) during LDL apheresis has been widely documented, supporting the pathogenetic role of CRP in atherosclerosis. CRP represents a state of chronic low-grade inflammation of the atherosclerotic arterial vessels and predicts cardiovascular outcomes. These findings are in line with what was also described by the JUPITER trial, where treatment with rosuvastatin was able to reduce not only LDL cholesterol, but also CRP, reducing the incidence of major cardiovascular events [27]. CRP levels are associated not only with inflammation but also with endothelial dysfunction and atherosclerosis progression, and the link is supported by the oxidative stress burden. In patients with essential hypertension, CRP and TNF-α correlated with 8-iso-prostaglandin-F2α, a marker of lipid peroxidation, characterized by vasoconstrictive and platelet-aggregation properties [28]. These data support the unique relation and the idea of a continuum between inflammation, oxidative stress, endothelial dysfunction and atherosclerosis progression.

Herchovici et al. found a significant decrease in CRP levels during LDL apheresis in hypercholesterolemic patients treated with three different LDL apheresis systems (DALI, DSA and plasma exchange) [29]. Similar findings have been described in other studies focusing on patients with PAD [30]. In addition, fibrinogen removal during LDL apheresis has been linked to reduced cardiovascular risk in patients with familial hypercholesterolemia [28]. This was confirmed by Kobayashi et al. in the setting of PAD, where fibrinogen levels decreased by around 24% compared with basal levels after 10 LDL apheresis sessions [31]. The degree of CRP and fibrinogen reduction may vary among the different devices used for LDL apheresis. Altogether, these data support the importance of such treatments for patients at high risk of prothrombotic events. Although there are no studies are available to date addressing the question of whether the modulation of such pro- and anti-inflammatory cytokine profiles in LDL apheresis is related to improved clinical endpoints, the impact of LDL apheresis in reducing inflammatory markers may be pivotal from a pathogenic point of view to mitigate the atherosclerosis process in high-risk patients.

### 3.2. Oxidative Stress and LDL Apheresis

CKD and ESRD patients are typically characterized by high levels of oxidative stress during the disease course [32]. Oxidative stress is characterized by an imbalance between reactive oxygen species (ROS) formation and/or their insufficient removal. Along with a proinflammatory condition, oxidative stress correlates with high levels of saturated fatty acids (SFAs) and, consequently, with the profound modulation of lipids and lipoprotein profiles in patients with CKD compared with healthy subjects, increasing the risk for atherogenesis and cardiovascular diseases [32]. An increasing body of evidence suggests a fundamental role of oxidative stress in promoting and aggravating endothelial dysfunction and cardiovascular disease as a result of the inhibition of endothelial nitric oxide synthase (eNOS) enzyme activity and reduced nitric oxide (NO) production and availability [33,34]. In addition, ROS inactivate both NO production and the renin–angiotensin system, leading to endothelial dysfunction and tubular damage [33]. Asymmetric dimethylarginine (ADMA), an analogue of L-arginine, is a naturally occurring product of metabolism found in the human circulation, and it is considered to be one of the predominant endogenous NOS inhibitors and causes of endothelial dysfunction [35]. Increased ADMA levels have been reported in patients with CKD and ESRD, mainly related to impaired degradation by the enzyme dimethylarginine dimethelaminohydrolase (DDAH) and reduced urinary excretion [33]. ADMA elevation is strongly associated with the dysfunction of the L-arginine/NO pathway, decreased NO production and endothelial dysfunction; in addition, from a clinical perspective, ADMA concentrations independently predict major adverse cardiovascular events (MACE, including myocardial infarction, coronary artery bypass graft, percutaneous coronary intervention, stroke, carotid revascularization and death) in patients with PAD [36]. The use of febuxostat, an inhibitor of xanthine oxidase enzyme, is effective in reducing the burden of oxidative stress and endothelial dysfunction in ESRD patients; in a randomized controlled trial including 57 ESRD patients, randomized to receive either oral 40 mg febuxostat or standard of care, febuxostat was able to significantly decrease serum malondialdehyde (MDA), as a marker of free radical activity, and increase superoxide dismutase (SOD) levels, as a marker of antioxidant enzymatic activity [34].

It has been demonstrated that LDL apheresis can lower LDL oxidation in patients with familial hypercholesterolemia [37], which is related to a significant and prolonged decrease in plasma hydroperoxide, total LDL cholesterol and polyunsaturated fatty acid concentrations. This increased resistance of LDL to oxidation was prolonged, supporting the idea of a long-term effect induced by LDL apheresis [37]. Yuko Tsurumi-Ikeya et al. have confirmed the prolonged effects of LDL apheresis on oxidative stress in patients on chronic hemodialysis, reporting a reduced level of circulating oxidized LDL, as a strong biomarker of lipid peroxidation [38]; moreover, the reduced plasma-oxidized LDL correlated inversely with walking distance, thus clearly impacting clinical outcomes [38]. Similarly, Haka et al. revealed that lipoprotein apheresis significantly decreased p22phox, an essential subunit of NADPH oxidase that can produce ROS, and p22phox gene expression [39]. In addition, levels of thiobarbituric acid-reactive substances (TBARS), a marker of lipid peroxidation, and ROS production were modulated by LDL apheresis [39]. Overall, these results strongly support the pathogenetic role of oxidative stress during PAD, and the ability of LDL apheresis to modulate ROS production and lipid peroxidation may represent a cornerstone in the therapeutic effects of such treatments in improving the progression of PAD and vascular complications. The prolonged effects on oxidative stress should be better analyzed with future well-designed longitudinal studies.

### 3.3. Endothelial Dysfunction and LDL Apheresis

Endothelial cells play a fundamental role in ensuring vascular protection against atherosclerosis. They have key roles in delivering nutrients and oxygen, regulating and maintaining adequate blood flow and tissue perfusion, and modulating immune cell trafficking, thus reducing the risk of progression to several cardiovascular conditions, such as ischemic heart disease, stroke and PAD [40]. The loss of endothelial protective functions is associated with the increased expression of adhesion molecules, such as E-selectin, P-selectin, intercellular adhesion molecule-1 (ICAM-1) and vascular cellular adhesion molecule-1 (VCAM-1). These molecules are mainly responsible for the attachment and rolling of leucocytes into the vessels, affecting endothelial integrity and function and, thus, inducing further endothelial damage and dysfunction. In addition, ADMA concentrations strongly correlate with oxidative stress and endothelial dysfunction, as previously mentioned; ADMA levels are independently related to endothelial-dependent vasodilatation (as assessed by flow-mediated dilation, FMD) and correlated with CKD stages [41]. Overall, these pathogenetic mechanisms are linked to the development of a prothrombotic phenotype of the endothelial cells, which increases the risk of atherosclerosis and reduces tissue perfusion [35]. Several studies have previously shown that LDL apheresis can improve endothelium integrity and function, enhance microcirculation (increase in nitric oxide synthesis with consequent vasodilatation), reduce hyperviscosity and platelet aggregation and, finally, reduce the amount of adhesion molecules in patients with familial hypercholesterolemia and CKD patients with PAD [42,43]. A single LDL apheresis session was able to significantly reduce E-selectin, VCAM-1 and ICAM-1 levels in patients with coronary arterial disease [44]. Similarly, Kobayashi et al. reported a significant reduction in P-selection after apheretic treatment in patients with PAD along with inflammatory markers and fibrinogen [31]. Utsumi et al. reported the effects on endothelial dysfunction in eight hemodialysis patients with PAD who underwent 10 weekly sessions of LDL apheresis with a dextran sulfate adsorber; after treatment, P-selectin, VCAM-1 and ICAM-1 were significantly reduced along with IL-1beta, IL-6 and TNF-α [45]. The effects of lipoprotein apheresis on microcirculation were also demonstrated in other specific clinical settings; Ramunni et al. reported the application of three sessions of LDL apheresis in a patient with severe acute anterior ischemic optic neuropathy (AION) with rapid and persistent improvement in their symptoms and visual acuity due to reduced papilla ischemia [46].

The specific molecular mechanisms involved in the effects of LDL apheresis on endothelial cells have been investigated. In vitro analyses using human umbilical vein endothelial cells (HUVECs) demonstrated that LDL apheresis increased the expression of activated endothelial nitric oxide synthase (eNOS) protein and induced increased and prolonged proliferative activity of HUVECs up to 3 months after treatment completion [38]. These effects are strongly impactful from a clinical point of view, as there was a statistically significant correlation between walking distance and the level of activated eNOS protein in HUVECs in the treated patients, demonstrating the clinical impact of such treatments [38].

### 3.4. LDL Apheresis Technique for PAD

Evidence on the application of lipoprotein apheresis in patients with PAD is limited and mainly based on observational studies with limited sample sizes. Poller et al. followed 10 patients with severe PAD and elevated levels of Lp(a) who had undergone previous revascularization procedures; the primary endpoint of the study was to analyze the rate of new procedures of revascularization in the following 12 months compared with the previous 24 months before apheresis; the secondary endpoints were related to the impact of such a treatment approach on the clinical symptoms of PAD (pain, walking distance) [47]. The efficacy of apheresis treatment was characterized by a lower frequency of revascularization required, along with a reduction in pain level estimated using specific scoring systems, and an increase in walking distance [47]. These data were also confirmed at the 2-year follow-up of this cohort, suggesting the long-term effects of lipoprotein apheresis in sustaining the improvement of microcirculation [48]. Similarly, a randomized clinical trial including 42 patients with primary hypercholesterolemia and coronaropathy randomized to receive LDL apheresis plus simvastatin or simvastatin alone demonstrated a significant reduction in the rate of major cardiovascular events (MACE) and lower limb stenosis in the apheresis group [49]. A combined treatment of LDL apheresis in addition to below-knee endovascular therapy (BK-EVT) in 25 patients on chronic hemodialysis with PAD showed a significant reduction in the rate of major amputations and re-interventions compared with patients who received only the endovascular treatment [50]. Another study including 31 patients with PAD reported significant improvement in the ankle brachial pressure index, walking distance and muscle microcirculation in patients receiving LDL apheresis, along with Fontaine stage improvement and the resolution of rest pain [51]. Focusing on the H.E.L.P. technique, Rietzsch et al. showed a significant effect of lipoprotein removal in 17 diabetic patients with septic foot lesions, showing a significant reduction in fibrinogen levels (dropped by 68%) after H.E.L.P. treatment; the clinical impact of treatment was significant, as only three patients unfortunately underwent major amputations and two patients received further surgical treatment [52].

The case report described above demonstrated the ability of H.E.L.P. apheresis to improve clinical outcomes when performed on top of standard treatment for PAD and in the absence of indications for further surgical procedures. At baseline, the patient did not present high levels of Lp(a) and LDL cholesterol, while he was characterized by fibrinogen levels above the upper limit. As shown in Table 1, the fibrinogen levels almost halve after a single session, returning to the baseline value before the next session; however, pre-treatment levels were lower during the first 2 months, when the patient underwent a more intensive treatment approach with weekly sessions. The rationale for this intensive approach in the first phase of treatment is based on several previous observations that supported the importance of the more pronounced removal of lipoprotein, fibrinogen and proinflammatory mediators in the early phase of PAD to impact clinical outcomes. In addition, the rapid recovery of fibrinogen and coagulative factors, as well as an increase in blood viscosity, soon after a single session has been documented. Conversely, a regular apheretic program may be useful to keep constantly low levels of the previously mentioned factors, attenuating thrombogenesis and atherosclerosis and improving perfusion and oxygen/nutrient supply at the tissue level. Future studies are required in order to define a specific and personalized protocol for H.E.L.P. apheresis, based on clinical (degree of vascular lesions at baseline) and laboratory (hypercholesterolemia, high levels of CRP and fibrinogen) findings; furthermore, a specific protocol for the maintenance phase should be set up, with the goal of keeping under control the key players in atherosclerotic progression (fibrinogen, inflammatory and endothelial markers, ROS) and reducing the risk of long-term complications. The significant improvements obtained after 6 months of treatment strongly suggest the potential for the widespread use of such a technique in this setting [12].

Although several pieces on evidence strongly support the use of lipoprotein apheresis as an adjuvant therapy for PAD, several questions still remain. The protocol adopted varies considerably between studies, and no consensus has been achieved regarding the management of the acute and chronic phases of the disease. The ASFA guidelines indicate a treatment course based on 1 to 2 sessions/week in the initial phase of treatment, followed by a transition to a single session every 2 weeks for a total of 5 to 10 procedures. However, a prolonged approach may be reasonable, particularly in chronic patients with severe and complicated vascular lesions [14]. Future studies are required to define an optimal protocol approach for such treatments in this specific setting. The volume of plasma treated should not be less than 3000 mL. The safety profile of the treatment has been widely documented; the use of angiotensin-converting enzyme (ACE) inhibitors is usually contraindicated in patients undergoing apheresis treatment, as the columns may induce plasma kallikrein generation followed by the conversion of bradykininogen to bradykinin. ACE inhibitors reduce the inactivation of bradykinin, and apheretic treatment during ACE inhibitor therapy may lead to intraprocedural complications, characterized by flushing and hypotension.

## 4. Conclusions and Future Directions

In conclusion, the case report described clearly documented the role of lipoprotein apheresis in inducing the reduction in prothrombotic and proinflammatory mediators, improving endothelial dysfunction, increasing peripheral tissue perfusion and reducing the risk of amputation in patients with severe PAD. The therapeutic effect of such a treatment depends on several mechanisms, including the reduction in oxidized LDL and oxidative stress, the reduction in proinflammatory cytokines and mediators, the reduction in fibrinogen and consequent reduced prothrombotic risk, and the improvement in microcirculation due to the activation of endothelial cells. In addition, new potential mechanisms may be implicated in such effects, and the recent availability of high-throughput technology may represent an innovative approach in this field in order to identify additional pathogenetic aspects of the role of LDL apheresis for treating PAD. Consistently, further well-designed studies are needed to assess specific and standardized protocols based on patients’ condition in order to treat patients with chronic comorbidities at high risk for PAD.

## Figures and Tables

**Table 1:** Trends in circulating lipids and fibrinogen during the treatment period.

	Intensive H.E.L.P. Treatment (Months 1–2)			Low-Intensity H.E.L.P. Treatment (Months 3–6)			Maintenance H.E.L.P. Treatment (from Month 7)		
	PRE	POST	REDUCTION	PRE	POST	REDUCTION	PRE	POST	REDUCTION
Total cholesterol (mg/dL)	134.2 ± 12.1	90.12 ± 7.1	44.2 ± 7.1	147.5 ± 9.6	100.7 ± 10.5	46.7 ± 10.6	144.6 ± 11.5	907.3 ± 9.7	47.2 ± 8.9
LDL cholesterol (mg/dL)	82.7 ± 12.3	45.2 ± 6.03	37.5 ± 7.9	93.8 ± 9.2	58 ± 7.5	35.8 ± 3.6	94.5 ± 8.6	50.3 ± 6.7	44.1 ± 7.4
HDL cholesterol (mg/dL)	37.5 ± 2.6	33.8 ± 1.9	3.66 ± 1.65	33.6 ± 2.72	29.7 ± 4.77	3.8 ± 2.3	41.3 ± 4.3	36.1 ± 3.6	5.2 ± 1.1
Triglycerides (mg/dL)	102.6 ± 26.8	55.1 ± 10.4	47.5 ± 19.5	163.6 ± 58.8	92 ± 47.8	70.3 ± 18.9	110.3 ± 47.6	67.8 ± 31.5	42.5 ± 19.9
Lp(a) (mg/dL)	23.5 ± 2.9	11 ± 1.5	12 ± 2.3	24.7 ± 3	11.78 ± 1.6	12.4 ± 2.9	21.4 ± 1.1	10	11.4 ± 1.1
Fibrinogen (mg/dL)	431 ± 50.1	231.6 ± 19.1	199.3 ± 40.5	510 ± 56.2	282.6 ± 25.7	228 ± 68.6	508.7 ± 63.4	254.8 ± 36.7	253.8 ± 65.9
CRP (mg/L)	11 ± 5	5.5 ± 1.5	5.5 ± 3.5	12.2 ± 3.5	5.7 ± 1.7	6.4 ± 1.7	19.8 ± 2	9.75 ± 0.8	10.1 ± 1.2

Data presented are related to the mean ± standard deviation of values obtained just before (pre), and after (post) apheresis sessions and the amount of reduction after treatment. Legend: CRP, C-reactive protein; HDL, high-density lipoprotein; LDL, low-density lipoprotein cholesterol; Lp(a): lipoprotein (a).
